# Abstracts from the Antimicrobial Resistance and Infection Control Conference 2018: Taif, Saudi Arabia. 13-15 March 2018

**DOI:** 10.1186/s13756-018-0334-0

**Published:** 2018-05-22

## Oral presentations

### OP-1 Role of effective communication in improvement of hand hygiene compliance in emergency department

#### Salwa Ghaiaty (sghiaty@moh.gov.sa)

##### Infection Prevention and Control Department, King Abdul Aziz Specialist Hospital, Taif, Kingdom of Saudi Arabia

**Background:** Hospital Acquired Infection (HAI) burden patients, complicate treatment, prolong hospital stay increase costs and can be life threatening.

Several studies showed that HCAI affect 4.6% to 9.3 % of hospitalized patients. Adequate Hand hygiene among health care personnel could prevent an estimated 15-30% of HCAI. Numerous studies over last few decades have shown that hand hygiene compliance rate is generally less than 50% of all opportunities Ineffective communication with staff or employee during transition of care is potential cause of non- compliance with hand hygiene practice by health care personnel.

**Aim of the study:** The study used was directed for effective communication with continuous feedback about performance.

**Methods:** As communication is the ability to convey information both effectively and efficiently. We combined multiple tools of communications. We started to use four types of effective communication in this study.

1. Visual communication, (a-highly visual passive screen saver, b-Tickers).
2. Verbal communication (Recorded video messages).
3. Non-verbal communication.
4. Written communication: (a- High impact alert messages, silent notification, do not disturb patient, b- Survey and quizzes for the staff).

**Results**: Improvement and increase hand hygiene compliance rate in ER reaching 74% after intervention. Compliance rate was 55% in October 2017 before implementing the study then it reached 64% after one month and recently it reached 74% in February 2018 with implementation of effective communication tools.

**Conclusion**: Simple measures to enhance communication through getting employee attention about the importance of hand hygiene by high impact alert messages, high visual screen saver, tickers survey and quizzes.

### OP-2 Penicillin resistance among *Streptococcus pneumonia* isolates at a tertiary care center in South West Saudi Arabia: five years retrospective study

#### Muhammad Halwani^1^, Muhammad Tyfor^2^

##### ^1^Department of Medical Microbiology, Al Baha University, Faculty of Medicine; ^2^Microbiology Laboratory, King Fahd Hospital., Al Baha, Kingdom of Saudi Arabia

###### **Correspondence:** Muhammad Halwani (mhalwani@bu.edu.sa)

**Background**: The emergence of different infections caused by penicillin-resistant strains of *Streptococcus pneumoniae* has become a worldwide concern. The magnitude of the problem in South West Saudi Arabia was not investigated.

**Methods**: Laboratory data on pneumococcal isolates was collected retrospectively from January 2010 to December 2014 from hospitalized patients in the main tertiary care hospital of Al Baha, South West Saudi Arabia. Minimum inhibitory concentrations (MICs > or = 2 mg/L) was used to detect the resistance isolates.

**Results**: During the five year duration, a total of 201 *S pneumoniae* isolates were identified, most of which (61%) 124/201 were isolated from respiratory specimens (sputum, tracheal aspirates, bronchoalveolar lavage), followed by eye swabs (15%) 30/201, blood (12%) 25/201, ear swabs (7%) 15/201 and CSF (3.4%) 7/201. The resistance rate of *S pneumoniae* was 71 % (43/60) on year 2010, 76% (35/46) on year 2011, 61% (22/36) on 2012, 68% (20/29) on 2013 and 66% (21/30) on 2014 respectively with an overall resistance of 70% (141/201).

**Conclusions**: The data confirm the presence of penicillin -resistant *S pneumoniae* in South West Saudi Arabia. This is in agreement with other studies that covered other parts of the country. The high resistance identified might indicate a potential concern and warn of further spread among individuals. Thus good penicillin control with periodical antibiotic surveillance may improve the current situation and attempt to slow down the accelerating problem of resistance to penicillin.

### OP-3 Withdrawn

## Poster presentations

### PP-1 Knowledge, attitude, safety practice of nutrition labels of university students

#### Shahida Shamsuddeen, Ayisha Ansari and Sara Al Rashidy

##### Clinical Nutrition, University of Hail, Kingdom of Saudi Arabia

###### **Correspondence:** Shahida Shamsuddeen (shahidaed1@gmail.com)

**Background:** There are limited studies conducted in Saudi Arabia to assess the knowledge, attitude and safety practice of the college students regarding nutrition labeling.

**Objectives:** The purpose of the study were to assess knowledge, attitudes and practice (KAP) about nutrition labeling among university students.

**Method:** A cross sectional survey was planned to assess knowledge, attitudes and practice (KAP) about nutrition labeling among students of University of Hail. The subjects were surveyed through a previously standardized self-administered questionnaire for questions related to their favorite food, nutrient content, knowledge, attitudes and practice of using nutrition labels. In addition, self-reported weight and height was collected and body mass index was calculated. The statistical analysis was done using Statistical Package for Social Sciences software (version 16.0).

**Results:** There was a significant difference (p < 0.05 or 0.000) between the mean scores of knowledge, attitude and practice between the nutrition and non-nutrition students. The nutrition students reported a very good knowledge on nutrient content (χ^2^=22.539, ORV-5.685), total calories (χ^2^=93.253, ORV-14.233), cholesterol (χ^2^=28.232, ORV-5.792), expiry date (χ^2^=7.901, ORV-0.850), food poisoning (χ^2^=11.355, ORV-14.235) stated in the nutrition labeling of the foods and was statistically highly significant(P<0.000). The attitudes of the nutrition students on reading information(χ^2^=12.816, ORV-15.881), making healthy food choice (χ^2^=12.729), taste (χ^2^=6.420, ORV-4.253), checking ingredients(χ^2^=10.835, ORV-6.006) and packaging(χ^2^=6.708, ORV-3.386), food allergies(χ^2^=9.499, ORV-7.197), food safety(χ^2^=9.562) were all found to be highly significant (P<0.01 or 0.000). The practices of nutrition students on reading list of ingredients (χ^2^=7.320), serving size(χ^2^=12.335), health claims(χ^2^=8.809), vitamins and mineral content (χ^2^=11.904), and checking the hygiene (χ^2^=8.717) was statistically significant (P<0.05, or 0.01).

**Conclusion:** Nutrition labels and its awareness were more useful tools for students and had an direct impact on the health status of the students. It is suggested to perform successfully designing, organizing and implementing the nutrition education programs in the institutions which will promote the level of knowledge about nutrition and nutrition labels thereby improving their food habits and lifestyle.

### PP-2 Antibiotics- loaded nanoparticles to treat drug resistant bacteria

#### Eman Halawani, Sanaa Fahmy and Seham Al Zahrani

##### Biology Department, Taif University, Taif, Kingdom of Saudi Arabia

###### **Correspondence:** Eman Halawani (Halawabi.em@hotmail.com)

There is worldwide concern about the rapid emergence of resistant bacteria and their resistance to commonly used antibiotics. Nowadays, the need for innovative strategies for developing antimicrobial drugs is becoming a necessity against antibiotic multi-resistant bacteria that have become of critical concern in public health. To overcome this multidrug-resistance problem, we studied for the first time a green, simple, and low-cost synthesis of silver nanoparticles using *Juniperus excelsa* leaves extract, to be used as alternative agents against multidrug-resistant bacteria. The synergistic effect of AgNPs with antibiotic was also studied. Multi-drug resistant bacteria were collected from Taif hospitals patients with skin infections. Phenotyping, biotyping and molecular characterization using 16S rRNA gene analysis of nonrepetitive multidrug-resistant (MDR) bacterial isolates were studied. The formation of later nanoparticles was characterized using FTIR, XRD, UV-VIS spectrophotometer and TEM. The antimicrobial studies were initially carried out by the determination of inhibition zone (ZIN), the minimal inhibitory concentration (MIC) and minimal bactericidal concentration (MBC). The biomolecules in the aqueous *Juniperus excelsa* leaves extract function as both safeties reducing and capping agents for biosynthesis of AgNPs. The shapes of AgNPs were spherical and hexagonal*.* The particle sizes ranged from 16.08-24.42 nm. The antimicrobial effect of AgNPs and their synergistic effect with antibiotic were studied against Methicillin- resistant *Staphylococcus aureus* (MRSA) and *Proteus mirabilis.* MICs of AgNPs against tested MDR bacteria ranged from 48-56 μg/ml, while MBCs of AgNPs against tested strains ranged from 72-96 μg/ml. Synergistic effects of AgNPs with cephalosporines, ampicillin and erythromycin, and showed 3 folds increase in the inhibition zone against tested strains. Consequently, we suggest that phyto-synthesized AgNPs are good alternatives in the treatment of diseases because of the presence of bioactive agents. Also, it showed a strong synergistic effect of AgNPs with antibiotic on tested bacteria. Consequently, it may be used to activate the antibiotics. These results indicate that AgNPs and AgNPs with antibiotic showed strong antibacterial properties on multi-drug resistant bacteria, which could be exploited in developing better dressings for wounds.

### PP-3 Assessment of the knowledge, attitude and practice about food safety among Saudi population in Taif

#### Ahmed Khalifa^1^, Khadiga Ismail^2,3*^, Farah Ansari^2^

##### ^1^Forensic and Toxicology Department, Faculty of Medicine, Ain-Shams University, Cairo, Egypt; ^2^Medical Laboratory Department, Faculty of Applied Medical Science, Taif University, Taif, Saudi Arabia; ^3^Parasitology Department, Faculty of Medicine, Ain-Shams University, Cairo, Egypt

###### **Correspondence:** Khadiga Ismail (Khadigaahmed68@yahoo.com)

Foodborne diseases outbreaks continue to be problem indicating the failure of population to adhere to safe practices during food preparation. Thus, this study aimed to assess the knowledge, attitude, and practices (KAP) of food safety awareness among public Saudi population. This study involved 136 persons from Taif Saudia Arabia. The food safety KAP among 136 Saudi population was assessed using a questionnaire. The study involved 54.4% females and 45.6% males, 56.6% work outside health field and 43.4% in health care field ,82.4% from urban area and 17.6% from rural area and 81% within the age group from 21-30 years old. 75.7% of the population had good attitude and practice towards health and food safety and washing hands before eating. Further population had low attitude on other related items such as unimportance of expire date in food safety 44.8% , unimportance of reading the instruction label on the caned food 59.5%, unimportance of checking the refrigerator temperature 77.9%, and unimportance of changing the cutting knife used between meat and vegetables cutting 66.9% . As regard knowledge , 61.8% of population had good knowledge about best temperature for bacterial growth which is between 4 to 50 °C and .About 73.5% of population had good knowledge about diseases that could be transmitted through food, but only 30.9% of population had knowledge about best method of meat thawing.

In conclusion, the suggestion of this study was that Saudi population had adequate food safety knowledge, but perceived knowledge failed to be translated into practices, therefore necessary to hold training programs through workshops or to include courses in the schedule of ministry of health.

### PP-4 Successfully control of methicillin resistance *Staphylococcus aureus* in neonatal intensive care unit in Taif, Saudi Arabia

#### Nabiha Bouafia^1^, Salha Alwegdani^1^, Samia Al Shehry^1^, Noha Abdulmotalib^1^, Mohammed Hamdi^1^, Mahmood Al Ashgar^1^, Timi Joseph^2^, Ali Al Zahrani^2^, Salwa Ghayati^3^, Sameerah AL Khaldy^3^, Malaka Amer^4^, and Waleed Mazi^1,5^

##### ^1^Department of infection prevention and control, King Faisal Medical Complex, Taif, Kingdom of Saudi Arabia; ^2^Department of neonatal intensive care unit, King Faisal Medical Complex, Taif, Kingdom of Saudi Arabia; ^3^Department of infection prevention and control, King Abdul Aziz Specialist Hospital, Taif, Kingdom of Saudi Arabia; ^4^Department of laboratory and blood bank, King Abdul Aziz Specialist Hospital, Taif, Kingdom of Saudi Arabia; ^5^Regional directorate for infection prevention and control, Taif, Kingdom of Saudi Arabia

###### **Correspondence:** Nabiha Bouafia (nabiha.bouafia78@gmail.com)

**Background:** Methicillin resistant *Staphylococcus aureus* (MRSA) is a critically important pathogen in neonatal intensive care units (NICUs) population and has been associated with both endemic and epidemic infections. *Staphylococcus aureus* nasal colonization is a well-known independent risk factor for infection.

**Purpose:** To decolonize neonatal MRSA organisms admitted to NICU at King Faisal Medical Complex, TAIF.

**Methods:** Eighty-three nasal swab samples were screened for MRSA during October – November 2017. *Staphylococcus aureus* were identified as round white colonies positive to Gram stain, catalase and slide agglutination tests. Methicillin-resistant phenotype was confirmed according to British Society for Antimicrobial Chemotherapy (BSAC) standards using the Vitek2 system (BioMerieux, USA). An isolate is considered methicillin resistant when the minimum inhibitory concentration (MIC) breakpoint of oxacillin is >2 mg/L and of cefoxitin >4 mg/L. MRSA decolonization was performed using nasal mupirocin ointment three times / day for 5 days. To confirm decolonization, cultures were taken after 2 days of stopped treatment. For prevention measures: hand hygiene, education program and cohort isolation were implemented during the study period.

**Results:** Eleven patients were colonized with MRSA and successfully decolonized after treatment with mupirocin ointment cream 2% for five days. Central line-associated bloodstream–MRSA infection was reported, treated with vancomycin, cured and discharged home.

**Conclusion:** Periodical assessment of MRSA is needed depends on the prevalence of hospital-onset-MRSA to control spread of organisms and maintain health and safe environment.

### PP-5 Evaluation of knowledge about HIV-infection and its relation to probiotics among female students of Hail University

#### Yomna Elkhateeb^1,2^, Noorah Alshammari^1^

##### ^1^Department of Clinical Nutrition, College of Applied Medical Science, University of Hail, Kingdom of Saudi Arabia; ^2^Microbial Chemistry Department, Genetic Engineering and Biotechnology Division, National Research Center, Cairo, Egypt

###### **Correspondence:** Yomna Elkhateeb (y.elkhateeb@uoh.edu.sa)

**Background:** Human immunodeficiency virus (HIV) *causes* acquired immunodeficiency syndrome (AIDS) by destroying “helper T cells”. In healthy individuals, helper T cells organize immune responses that protect the body from infection. Survival rates among patients have improved since the introduction of antiretroviral therapy (ART) in HIV management. Studies suggests that supplementation of prebiotics and probiotics is beneficial for (ARV)-treated HIV patients.

**Aim:** Objective of study is to assess knowledge of female students of Hail University about probiotics, prebiotics and their effective role with antiretroviral drugs in treatments of HIV patients. Also, provides knowledge about probiotics, prebiotics and their relationship to HIV disease.

**Method:** A cross sectional survey was planned to evaluate knowledge about HIV-infection and its relation to probiotics among female students of Hail University through a previously standardized self- administered questionnaire. Then, comparing degree of knowledge between literature and scientific colleges.

**Result:** there is a highly significant differences among levels of knowledge (Excellent 3%; Very good 13% ; Good 20%; Pass 43% and Fail 21%) at P < 0.05 or P < 0.01. Statistical analysis for the degree of knowledge showed that there is a high significant differences in degree of knowledge between students of literature colleges (58.93± 11.73) and students of scientific colleges (73.20 ± 9.08). Results indicate that students of scientific colleges have higher level of knowledge than literature colleges at P < 0.05 or P < 0.01.

**Conclusion:** Study provides an update and a discussion on the influence of prebiotics and probiotics in the management of HIV infections. The results provide perspective for discussing potential future therapeutic strategies focused on modulating the gut microbiota to abate HIV disease progression. These findings should encourage consumption of fermented dairy products and vegetables & fruits as part of the total daily food consumption. That they contain probiotics and prebiotics.

### PP-6 Antibiotic resistance among clinical *Klebsiella* isolates in 2010 and 2017

#### Nesma Mousa^1^, Moustafa El-Shenawy^2^ and Lobna El-Hosseiny^3^

##### ^1^Department of Environmental Studies, Institute of Graduate Studies and Research, Alexandria, Egypt; ^2^Department of Food Microbiology, National Research Center, Dokki, Cairo, Egypt; ^3^Department of Environmental Studies, Institute of Graduate Studies and Research, Alexandria, Egypt

###### **Correspondence:** Nesma Mousa (nesmanasser@yahoo.com)

**Background:** *Klebsiella* species are found everywhere in nature leading to a wide range of diseases including pneumonia, septicemia, meningitis and soft tissue infections. Recently, pathogens have developed resistance to antibiotics posing serious threats to human health globally.

**Objectives:** This work highlights comparatively the incidence and pattern of antibiotic resistance for clinical *Klebsiella* isolates in 2010 and 2017.

**Methods:** Fifty-five *Klebsiella* isolates (20 in 2010 and 35 in 2017), from blood of patients admitted to ICU, were tested for their sensitivity to fifteen antibiotics including Amoxicillin/ Clavulanic acid, Ampicillin/ Sulbactam, Piperacillin/ Tazobactam, Aztreonam, Meropenem, Imipenem, Cefotaxime, Ceftriaxone, Ceftazidime, Cefoperazone/Sulbactam, Amikacin, Tobramycin, Gentamicin, Trimethoprim/Sulfamethoxazole and Doxycycline.

**Results:** In 2010, the resistance percentages to the tested antibiotics ranged from 0-30%, however this percentage increased by 2-3 folds in 2017 especially towards tobramycin, cefotaxime, ceftriaxone, ceftazidime, piperacillin/tazobactam, cefoperazone/sulbactam and ampicillin/sulbactam.

**Conclusion:** The change in pattern and incidence of resistance among pathogens is alarming requiring collaborative efforts among health sector partners to combat this phenomenon.

### PP-7 Pattern and risk factors of sharp object injuries among health care workers in tertiary hospitals

#### Nuha Abd Elmotalab^1^, Sitalnesa Abdelhafeez^2^, Raja Fadil^3^, Nabiha Bouafia^1^, Mohammed Abdelwahab^1^, Arlene Samson^4^ Mahmoud Al Ashgar^1^Muznah AL Shalawi^3^ Sultan Al Ghamdi^3^, Saleh Al Zahrani^3^ and Waleed Mazi^1^

##### ^1^Infection Prevention Control Department in King Faisal Medical Complex, Taif, Kingdom of Saudi Arabia; ^2^Department of Medical Education and Research Unit, King Abdul Aziz Specialist Hospital, Taif, Kingdom of Saudi Arabia; ^3^Department of Public Health, King Abdul Aziz Specialist Hospital, Taif, Kingdom of Saudi Arabia; ^4^Department of Employee Health Clinic, King Faisal Medical Complex, Taif, Kingdom of Saudi Arabia

###### **Correspondence:** Nuha Abdelmotalab (Noha_hamoda@hotmail.com)

**Background:** Accidental occupational exposure of healthcare workers to blood and body fluids after skin injury constitutes a risk for transmission of blood-borne pathogens.

**Aim:** To assess pattern and risk factors of sharp object injuries in two tertiary hospitals (King Abdulaziz Specialist hospital and King Faisal Medical Complex Saudi Arabia –Taif.

**Methods:** Retrospective review of registry records from staff clinic was used to collect data including all employees exposed to sharp object injuries during 2016 – 2017.

**Results:** A total of 131 employees were exposed to sharp object injuries during 2016 -2017. The incidence increased from 2.89 /10.000 patient days in 2016 to 3.422/ 10.000 patient-days in 2017. From total number 131, Females 98(74.8%) were affected more than male 33 (25.2%). Mean age was 31 ± 6.6. Most affected employee were young (20-30years constitute 73 (55.7%). Most injuries occurred in Emergency room 26 (19.8%) followed by Surgical word 20(15.3%), OR 16(12.2%). Nurses were more affected 74(56.5%), doctor 23(17.6%) housekeeping 18 (13.7%). According to type of exposure high exposure due to needle prick 104 (79.4%), cut wound 15(11.5%). Most devices caused injuries were bore hollow needle 63(48.1%), suture needle 18 (13.7%), cannula and insulin syringe each 13 (9.9%). Regarding circumstances of injury, most during operation 23 (17.6%) , during waste collection 15 (11.5%) , cannulation 12 (9.2%) and giving injection 12 (9.2%) . Exposure to HBV, HCV and HIV were 4(3.1%), 3(2.3%) and 3 (2.3%) respectively. No seroconversion among employee was documented.

**Conclusion:** There is increase in incidence of sharp needle injuries from 2016 to 2017.Female were more exposed than males. The younger and nurses were more affected. Emergency department was the most affected area. Bore hollow needle was the most device causing sharp injuries. Sharp injuries during operation were the most circumstances during which exposures occurred followed by waste collection.

### PP-8 Incidence of healthcare associated infections in acute care hospitals in Taif, Saudi Arabia

#### Masood Shah^1^, Mohammed Hamdi^2^, Mahmood Al Ashgar^2^, Salwa Ghayati^3^, Abdullah Al Qarni^4^, Avigail Tan^3^, Najla Helali^3^, Yuvon Aldecoa^2^ , Samah Al Homaidi^4^ and Waleed Mazi^1,2^

##### ^1^Regional Directorate for Infection Prevention and Control, Taif, Kingdom of Saudi Arabia; ^2^Infection Prevention and Control Department, King Faisal Medical Complex, Taif, Kingdom of Saudi Arabia; ^3^Infection Prevention and Control Department, King Abdul Aziz Specialist Hospital, Taif, Kingdom of Saudi Arabia; ^4^Infection Prevention and Control Department, Children's Hoaspital, Taif, Kingdom of Saudi Arabia

###### **Correspondence:** Masood Shah (mashahnawaz@moh.gov.sa)

**Background:** Health care-associated infections (HAIs) increase mortality, length of hospital stay, costs of care, bacterial resistance, antibiotic usage, and other adverse events. Monitoring HAI indicators is one of patient safety component. We describe the distribution of HAIs in three hospitals in Taif, Saudi Arabia.

**Methods:** A retrospective study was conducted on three acute care hospitals during the year 2017. Patient population, surveillance, calculation of incidence rate and ratio, benchmarking and interpretations were conducting according to National Safety Healthcare Network (NHSN) USA.

**Results:** Incidence rates of ventilated associated pneumonia (VAP) were high (> 90 percentile) with median utilization ratio (50-75 percentile) followed by catheter urinary tract infections (75-90 percentile) with median utilization ratio (50-75 percentile) in adult intensive care units (ICU). Incidence rate of central line associated bloodstream infections (CLABSI) was high (>90 percentile) despite of low utilization ratio (10-25 percentile) in adult ICU of post graduate teaching hospital. However, the incidence rate of CLABSI was (25-50 percentile) with the same utilization ratio (25-50 percentile) in ICU of graduate teaching hospital. Incidence rate and ratio of VAP, CAUTI and CLABSI were high (>90 percentile) in medical/surgical department of post graduate teaching hospital. Incidence rate of CLABSI and CAUTI were high (> 90 percentile) with low utilization ratio in ICU and Medical/Surgical department of pediatric non- teaching hospital. Incidence rates of Appendix (APPY) and cesarean section (CSEC) infections were low (0.9% and 0.64%, respectively.

**Conclusion:** Action plan is needed to reduce VAP and CLABSI in all hospitals. Surveillance should be revised and improved to avoid underestimated HAIs. Chronic patients should be excluded from the medical/surgical departments.

### PP-9 Molecular characterization of antibiotic resistant *E. coli* isolated from UTI patients

#### Fethi Ben Abdallah^1^, Badriah Al_ Sarhan^1^, Waleed Mazi^2,3^ and Rihab Lagha^1^

##### ^1^Department of Biology, Faculty of Science, Taif University, Taif, Kingdom of Saudi Arabia; ^2^Unité de Recherche : Virologie & stratégies antivirals, Higher Institute of Biotechnology, Monastir. Tunisia; ^3^Infection Prevention and Control Department, King Faisal Medical Complex, Taif, Kingdom of Saudi Arabia

###### **Correspondence:** Fethi Ben Abdallah (fetyben@yahoo.fr)

Antibiotic therapy is one of the most important therapies used for fighting infectious diseases and has tremendously enhanced the health aspects of human life since its introduction. Despite the advancements in this therapy, we still live in an era where incidents of antibiotic resistant infections are alarmingly on rise.

In this work, 50 clinical *Escherichia coli* were isolated from urine of UTI patients in King Abdulaziz hospital, Taif city, KSA. Their ages ranging from two months to 90 years containing 31 females, 12 males and 7 children. Antibiotic susceptibility of isolates was tested on 25 antibiotics. Our finding showed a resistance to all antibiotic except Meropenem. The percentage of resistance was ranged from 100% for penicillin to 2% for imipenem. In addition, 30% of the isolates appeared as for Extended Spectrum Beta-Lactamase (ESBL) positive. Out of the 50 isolates we noted theexistence of 46 profiles and 74% of isolates are considered as multi-drug resistance strains. Distribution of antibiotic resistance genes realized by Polymerase Chain Reaction (PCR) showed that the genes *aac(3)-IV* and *bla*SHV were identified in 33.33% of isolates. In addition, the genes *qnrA*, *bla*CMY and *dfrA1* were founded in 37.25%, 19.60% and 17.64% of the isolates respectively. In total, 17 different genotypes were detected and 12 isolates (24%) do not include any genes in their genomes.

### PP-10 Hand hygiene practices among medical students

#### Nesreen Bakhsh (nosa1402@gmail.com)

##### Department Of Laboratory, King Abdul Aziz Specilist Hospital, Taif, Kingdom of Saudi Arabia

***Background*:** Hand hygiene is a cost-effective method in preventing infection transmission. Hand hygiene practices have been found to be faulty in most healthcare settings. We conducted a study to evaluate the awareness, and compliance of hand hygiene among undergraduate medical students during their clinical phase in Taif University Medical College, Saudi Arabia.

**Methods:**A questionnaire based on World Health Organization's concept of “Five Moments for Hand Hygiene” was used to evaluate the awareness of the indications for hand hygiene and compliance was observed during Objective Structured Clinical Examination (OSCE) sessions. Sixty students including thirty-six males (60%) and twenty-four females (40%) participated voluntarily in the study.

**Results:** The average awareness regarding the positive indications of hand hygiene was 56%. Rest of the 44% of students was either not sure or unaware of the indications of hygiene. Only 29% of students were able to identify all the five indications for hand hygiene in the questionnaire. Compliance as assessed during OSCE sessions was only 17% with no significant difference between the genders.

**Conclusion:** It was concluded that serious efforts are needed to improve the hand hygiene practices among medical students.

### PP-11 Antibiotic resistance of *Escherichia coli* Strains isolated from animal original food in 2007 and 2015

#### Mohammed Fouad, Moustafa El-Shenawy

##### National Research Center, Department. Dairy & Food Microbiology, Dokki - Cairo, Egypt

###### **Correspondence:** Mohammed Fouad (elshenawy_moustafa51@yahoo.com)

**Introduction:** *Escherichia coli* is commonly found in intestinal tracts and, as a result of fecal contamination or contamination during food animal slaughter, is often found in foods of animal origin.

**Objectives:** This study was conducted to determine the antibiotic sensitivity pattern of *E. coli* isolated from different types of animal original foods collected randomly from street-vendors and super-markets in Giza.

**Methods:** Thirty-one isolates of *Escherichia coli* recovered in 2007 and twenty-seven isolated recovered in 2015 were examined to better understand the prevalence of antimicrobial resistance among these organisms. Antimicrobial susceptibility testing was performed using 15 antimicrobials including chloramphenicol, ampicillin, amoxicillin-clavulanic acid, cephalothin, ceftiofur, ceftriaxone, gentamicin, sulfamethoxazole, trimethoprim-sulfamethoxazole, nalidixic acid, ciprofloxacin, tetracycline, and cefoxitin. The antibiotic susceptibility test was performed using the agar disk diffusion test.

**Results:** The results indicated that. There was an increase in the frequency of resistance to a majority of antimicrobials tested. The overall incidence of drug resistant *E. coli* was 18% in 2007 increased to 62% in 2015. Extended Spectrum β-Lactamase (ESBL) producers were more detected in 2015.

**Conclusion:** Multidrug resistant strains of *E. coli* are a matter of concern as resistance genes are easily transferable to other strains in the human and might pose a potential health risk to the consumer. Therefore, in order to avoid this, good hygienic practices during handing and processing of these foods are necessary.

### PP-12 Molecular characterization of carbapenem resistant Enterobacteriaceae from intensive are units of a tertiary care hospital od Islamabad, Pakistan

#### Azka Fatima, Rubine Kamran, Haniya Rashid and Muhammad Shafique

##### Pakistan Institute of Medical Sciences, Islamabad, Pakistan

###### **Correspondence:** Azka Fatima (azkafatimadub@yahoo.com)

**Background:** Enterobacteriacea are gram negative rods causing serious infections in intensive care units (ICUs) of hospitals. They are glucose fermenter, catalase positive and oxidase negative. These organisms are showing resistance to several classes of antimicrobials and resistance genes are spreading by acquired plasmids in bacterial population. Resistance to carbapenem group of antimicrobials is an emerging problem for clinicians and surgeons. Because after this class of antibiotics, no other treatment regimen is left workable to treat infections.

**Methods:** This research was a descriptive, cross sectional study. A proforma was used as a tool for data collection. Eighty three isolates of CRE from intensive care units samples were processed in 9 months duration from July 2015 to March 2016.This study was conducted in department of Pathology, Microbiology, Shaheed Zulfiqar Ali Bhutto Medical University, Pakistan Institute of Medical Sciences, Islamabad.

Data was collected through samples coming from ICUs. And organisms were cultured on blood and Mac Conkey agar. Organisms were identified, confirmatory tests were done and sensitivity of drugs was checked by Kirby Bauer disc diffusion method according to CLSI manual 2014. Resistant organisms to imipenem and meropenem were preserved in glycerol broth at -800C. MIC of imipenem was checked by using E-strips. Their molecular characterization was done using the conventional PCR targeting NDM, KPC, VIM and IMP genes.

**Results:** Minimum age of patient was 2nd day of life while maximum age of patient was 80 years of life.There were 36 (43%) females and 47 (57%) males. Out of 83 samples, 22 (26.5%) were from urine, 22 (26.5%) were from endotracheal tube tip, 12(14%) were from blood, 11 (13%) were from pus, 11 (13%) were from tracheal secretions, 3(4%) were from fluids and 2(3%) were from catheter tip.

In this study, out of 83 samples of CRE,62(75%) were klebsiella pnemoniae, 14(17%) were e.coli, 2(2.25%) were klebsiella specie, 2(2.25%) were enterobacter agglomerans, 2(2.25%) were enterobacter cloacae and 1(1.25%) were klebsiella oxytoca.

From total of 83 samples isolated from intensive care units, 83(100%) were found sensitive to Tigecycline, and Polymxine B 34 (41%) were sensitive to Chloramphenicol, 14 (16.9%) were sensitive to Aztreonam, 12(14.5%) were sensitive to Amikacin. There were 0(100%) resistance to Imipenem, Meropenem, Ertapenem, Amoxicillin+Clavulanic acid, Ceftriaxone, Ceftazidime, Tazobactam+Pipercillin, Ciprofloxacin, Gentamicin,

Tobramycin, Sulfamethoxazole+Trimethoprim, Nitofurantoin, Nalidixic Acid. The minimum inhibitory concentration of imipenem were between ranges of 4 μg/ml to more than 32 μg/ml. NDM was isolated in 56% isolates. VIM, KPC, IMP was not found in any study isolates.

**Conclusion:** CRE are 100% resistant to Imipenem, Meropenem, and Ertapenem in this study. Tigecycline and polymxin B are parental drug which is found effective against CRE isolates.14.5% of CRE isolates were sensitive to Amikacin in our study. MIC of imipenem showed 100% resistance for CRE isolates. NDM gene was present in 56% samples. Whereas VIM gene, KPC gene, IMP genes were not detected in our CRE samples. NDM positive isolates were 48% *Klebsiella pneumoniae.*

